# Marine Algae and Deriving Biomolecules for the Management of Inflammatory Bowel Diseases: Potential Clinical Therapeutics to Decrease Gut Inflammatory and Oxidative Stress Markers?

**DOI:** 10.3390/md22080336

**Published:** 2024-07-25

**Authors:** Alberto Repici, Ahmed Hasan, Anna Paola Capra, Sarah Adriana Scuderi, Irene Paterniti, Michela Campolo, Alessio Ardizzone, Emanuela Esposito

**Affiliations:** 1 Department of Chemical, Biological, Pharmaceutical and Environmental Sciences, University of Messina, Viale Ferdinando Stagno D’Alcontres, 31, 98166 Messina, Italy; alberto.repici@unime.it (A.R.); ahmed.hasan@studenti.unime.it (A.H.); annapaola.capra@unime.it (A.P.C.); sarahadriana.scuderi@unime.it (S.A.S.); ipaterniti@unime.it (I.P.); campolom@unime.it (M.C.); eesposito@unime.it (E.E.); 2 School of Advanced Studies, Center of Neuroscience, University of Camerino, 62032 Camerino, Italy

**Keywords:** inflammatory bowel disease (IBD), marine algae, inflammation, oxidative stress

## Abstract

The term “inflammatory bowel disease” (IBD) describes a class of relapse-remitting conditions that affect the gastrointestinal (GI) tract. Among these, Crohn’s disease (CD) and ulcerative colitis (UC) are two of the most globally prevalent and debilitating conditions. Several articles have brought attention to the significant role that inflammation and oxidative stress cooperatively play in the development of IBD, offering a different viewpoint both on its etiopathogenesis and on strategies for the effective treatment of these conditions. Marine ecosystems may be a significant source of physiologically active substances, supporting the search for new potential clinical therapeutics. Based on this evidence, this review aims to comprehensively evaluate the activity of marine algae and deriving biomolecules in decreasing pathological features of CD and UC. To match this purpose, a deep search of the literature on PubMed (MEDLINE) and Google Scholar was performed to highlight primary biological mechanisms, the modulation of inflammatory and oxidative stress biochemical parameters, and potential clinical benefits deriving from marine species. From our findings, both macroalgae and microalgae have shown potential as therapeutic solutions for IBD due to their bioactive compounds and their anti-inflammatory and antioxidant activities which are capable of modulating markers such as cytokines, the NF-κB pathway, reactive oxidative and nitrosative species (ROS and RNS), trefoil factor 3 (TFF3), lactoferrin, SIRT1, etc. However, while we found promising preclinical evidence, more extensive and long-term clinical studies are necessary to establish the efficacy and safety of marine algae for IBD treatment.

## 1. Introduction 

Inflammatory bowel disease (IBD) principally encompasses two well-known chronic inflammatory conditions of the gastrointestinal tract: Crohn’s disease (CD) and ulcerative colitis (UC) [1]. Both pathologies are characterized by an abnormal immune response, which leads to the rise of an inflammatory status in different parts of the intestinal tract. Indeed, CD usually impacts the end of the small intestine and the beginning of the colon while UC, on the other hand, affects the colon and, especially, the rectum [1]. 

Although the exact cause of IBD remains unclear, it is believed that these disorders result from a combination of genetic predisposition and environmental factors that, in association with the immune system, alter the physiological gut homeostasis [2]. All this leads to a series of remitting-relapsing gastrointestinal symptoms including persistent diarrhea, abdominal pain, rectal bleeding, weight loss, and fatigue [3]. 

The diagnosis of IBD typically involves a combination of endoscopic procedures, imaging studies, and laboratory tests to assess the inflammation and exclude other conditions [4]. Currently, the pharmacological treatment of this disease aims to reduce inflammation and achieve and maintain remission, without, however, leading to a complete recovery [5]. The therapeutic regimen can include the administration of aminosalicylates, corticosteroids, immunomodulators, and, recently, also biological therapies [6]. In the most severe cases, surgery may be necessary to remove damaged sections of the gastrointestinal tract [7].

Living with IBD can be difficult, requiring ongoing medical care and lifestyle changes, so patients often need to modify their dietary intake, manage stress, and receive regular medical checkups. Despite these challenges, many IBD subjects lead full and active lives with appropriate management and support. 

Research continues to advance in understanding IBD, offering hope for more effective treatments and, ultimately, aiming to find a resolutive cure. In this regard, several articles have highlighted the crucial roles of inflammation and oxidative stress in the pathogenesis of IBD [2,8]. Indeed, the persistent inflammatory response and oxidative stress overbalance induce the inappropriate immune reaction and the dysbiosis of intestinal flora, also leading to the release of pro-inflammatory cytokines and other harmful mediators [9]. As a consequence, cytokines perpetuate the cycle of inflammation, increasing the extent of tissue injury and IBD symptomatology [10]. 

While the role of inflammation in IBD has been widely investigated, the role of oxidative stress is still the object of further investigation.

Oxidative stress refers to an imbalance between the production of reactive oxygen species (ROS) and the body’s ability to detoxify these harmful compounds through antioxidants’ endogenous defense [11,12]. In IBD, excessive ROS production occurs due to chronic inflammation, overwhelming the antioxidant system and thus altering the physiological balance of the patient [13]. Such an oxidative stress status further exacerbates intestinal damage by attacking cellular components such as lipids, proteins, and DNA, overall resulting in a negative loop where inflammation increases oxidative stress and vice versa [13].

Recent clinical studies have shown that IBD patients displayed higher levels of oxidative markers and lower levels of antioxidant species in their intestinal tissues, corroborating that this imbalance could contribute to the pathophysiology of IBD [14,15]. The importance of addressing both inflammation and oxidative stress in treatment strategies, antioxidants, and anti-inflammatory agents are being explored to break this cycle and offer new therapeutic benefits.

The understanding of the interplay between inflammation and oxidative stress can open new avenues for scientific research to effectively manage symptoms as well as prevent long-term IBD complications. 

In this context, seaweed has emerged as a promising area of research for potential therapies in the treatment of IBD [16]. These marine plants are rich in bioactive compounds such as polysaccharides, polyphenols, and essential minerals, which possess numerous anti-inflammatory, antioxidant, and immunomodulatory properties 17. Hence, considering that chronic inflammation and oxidative stress are involved in IBD, seaweed may offer a natural and alternative approach to treating these pathologies.

Clinical studies investigating the effects of seaweed supplementation in IBD patients are still in the early stages of assessing potential improvements in clinical symptoms, inflammatory markers’ reduction, and the enhancement of antioxidant capacity. Overall, the diverse bioactive properties of seaweed make it a potential candidate for natural IBD treatment. As research progresses, seaweed-based therapies could become a valuable addition to conventional treatments, offering a natural, holistic approach to managing this complex condition. 

This review aims to investigate the potential benefits of marine algae, or the biomolecules produced from them, in treating IBD. Specifically, we examined the literature to report the biological activities of seaweed in IBD pathological settings as well as to evidence the potential modulation of clinical parameters associated with oxidative stress and inflammatory markers.

## 2. Search Strategy

### Methods

A literature search was conducted using the PubMed (Medline) and Google Scholar bibliographic databases. Only high-quality studies that examined the use of marine algae in preclinical or clinical models of IBD, up until April 30, 2024, were included. Relevant terms related to marine algae and IBD were searched using specific keywords in the database, as specified below:

Marine algae: marine algae; seaweed; macroalgae; microalgae; brown algae; red algae; green algae; blue-green algae.

IBD: inflammatory bowel disease; ibd; crohn’s disease; ulcerative colitis; colitis; gastrointestinal inflammation; chronic inflammation; intestinal inflammation; ibd flares; ibd treatment; immune-mediated inflammatory diseases; ibd symptoms; bowel disease; intestinal disorders; ibd management; ibd therapies; ibd biomarkers; mucosal healing; gut microbiota; autoimmune disease; gastroenterology; ibd clinical trials.

## 3. Pathophysiology of IBD

Multiple factors contribute to the complexity of IBD, making the pharmaceutical treatment of these disorders challenging [18]. These illnesses often manifest between the ages of 15 and 30 [19], although, in many cases, symptoms can start to appear at an earlier age or even at birth [20]. Over the past few decades, there has been a rising trend in the incidence of IBDs. The prevalence of IBD was 321.2 per 100,000 people in 2021; this is a + 46% increase in prevalence from 2006, when it was 200 per 100,000 people [21]. This was first seen in Western nations and is currently becoming more widespread globally [22]. IBDs are acknowledged as a significant global public health concern with rising financial and economic burden [23]. The scientific community generally agrees that a genetic predisposition is required for the development of IBDs, as demonstrated by the mutation of the gene nucleotide-binding oligomerization domain-containing protein 2 (NOD2) associated with CD [24]. The risk factors are not only genetic but include environmental factors, as evidenced by the higher incidence of IBDs in urban areas compared to rural [25], and including lifestyle habits such as smoking or a high intake of sugar in the diet [26,27]. However, intestinal dysbiosis remains the most well-recognized cause of IBDs [28]. As stated, the distinction between UC and DC impacts the affected region of the gastrointestinal system and the disease’s symptomatology. The major symptoms of UC are colon inflammation, with the possibility of flare-ups involving fever, cramping in the abdomen, and hemorrhagic diarrhea [28]. The pathophysiology of IBDs is quite complex and, in addition to the susceptibility of certain individuals, it is important to also consider the behavior of the immune system in the intestine [29]. For instance, molecular patterns associated with damage (DAMP) and molecular patterns associated with pathogens (PAMP) are two types of ligands that the pattern recognition receptors (PRR) identify [30]. These ligands are important in the development of IBDs. Certainly, an excessive innate and adaptive immune system response to normal intestinal microorganisms rapidly worsens patients’ conditions [31]. Thus, in the first state of IBDs, inadequate resolution of the inflammatory response to the initial insult keeps the illness recurring [32]. The immune system and IBD appear to be closely related; a weak immune system that is unable to counter microbial activity or fully repair wounds eventually results in IBD [33]. Neutrophils, which are directly recruited from the circulatory system into the inflamed region of the gastrointestinal tract, typically get involved as a result of an infection or damage to the intestinal mucosa [34]. How proinflammatory and immunosuppressive factors interact can influence how inflammation evolves during IBDs [35]. When exposed to diverse antigenic stimuli, macrophages release cytokines, which bind to various receptors and have endocrine, paracrine, and autocrine effects. Lymphocytes are differentiated into several T cell types by cytokines. Type 1 (Th-1) helper T cells are mostly linked to CD, while Type 2 (Th-2) helper T cells are primarily linked to UC [36]. The uncontrolled immunological reaction causes an ongoing inflammatory process by upsetting the gut mucosa, but it is important to keep in mind that T cells do not proliferate in the mucosa but invade the local site of inflammation through blood circulation [37]. Since neutrophils cause the generation of ROS, which directly harms the gastrointestinal tract’s epithelial barrier, they appear to have significant involvement in IBD [38]. A disproportionate enzymatic response that produces a variety of inflammatory agents is caused by the concentration of hyperactivated neutrophils, and this response alters the structure of Lieberkühn crypts and promotes the development of cryptic abscesses [39]. 

Furthermore, one of the main characteristics of both UC and CD is the depletion of goblet cells, which are essential for maintaining the digestive system’s homeostasis since they release protective mucous secretions [40]; neutrophils can also accelerate the depletion of goblet cells [41]. Therefore, it is evident that there are several triggers and contributing variables to the pathophysiology of IBD that are at the crossroads between inflammation and oxidative stress. Thus, it is crucial to keep researching IBD while accounting for all these diseases’ different components.

**Figure 1:**
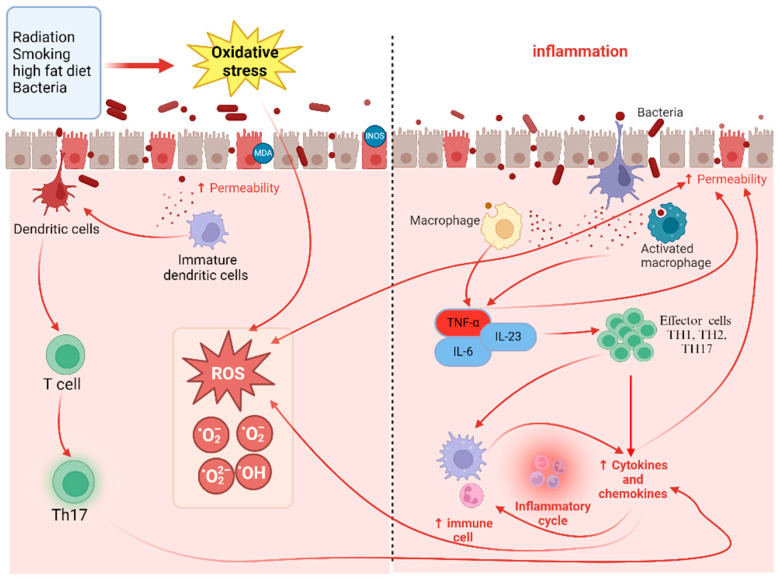
Schematic representation of link between oxidative stress and inflammation in inducing IBD, illustrating how external factors, such as diet, smoking, environmental triggers, and genetic mutations induce inflammation and oxidative stress, resulting in gut dysbiosis and damage to the intestinal barrier. This damage activates an inflammatory response characterized by the release of pro-inflammatory cytokines and chemokines, which perpetuates oxidative stress and inflammation.

A summary of oxidative stress and inflammatory triggers of IBD is provided in Figure 1.

## 4. Inflammatory and Oxidative Stress Markers Related to IBD

### 4.1. Cytokines Related to the Inflammatory Response in IBD

The interaction between immune, epithelial, and mesenchymal cells in managing intestinal inflammation involves intricate interactions among cytokines and chemokines. 

Clinical trials using TNF-α blockers have shown that cytokines are effective therapeutic targets for IBD patients. TNF-α is known to induce apoptosis in activated inflammatory cells. Infliximab, a monoclonal anti-TNF-α antibody, is widely used to treat acute and subacute CD [42]. TNF-α is a useful marker of disease activity in children and needs more investigation for adult patients [43].

The fecal pro-inflammatory cytokines, such as IL-1β and IL-6, are increased in patients with active IBD, reflecting the mucosal inflammation, while IL-2 and IFNγ are down-regulated in the peripheral blood mononuclear cells of UC and CD patients [44,45]. In chronic inflammatory autoimmune diseases, including IBD, there is a defect in regulatory T cells, which maintain the balance between the Th1 and Th2 immune responses. In the case of IBD, Th1 cells predominate, and are responsible for the production of IFN-γ, IL-2, and TNF-β [46]. Studies have shown a direct link between the level of IL-6 in the serum and the disease severity of inflammatory bowel disease [47]. IL-6 and IL-23 work synergistically to activate Th17 cells. In mouse models of enterocolitis and colitis, IL-23-activated Th17 cells were found to produce significant amounts of IL-17 and IL-6, indicating a synergistic action of these cytokines in the inflammatory process [48]. 

Recent research highlights the significant contribution of IL-17 and IL-23 in developing chronic intestinal inflammation. IL-23 plays a crucial role in promoting the development and expansion of a pathogenic T-cell population, which is responsible for producing IL-6 and IL-17 [49,50]. Treating intestinal lymphocytes with IL-23 enhances the IL-17A production in UC and IFNγ production in CD, indicating that the Th1/Th17 balance in IBD is regulated by IL-23 [51]. Transforming growth factor-β1 (TGFβ1), which is an inhibitory cytokine, and IL-10 are both essential for maintaining immunological homeostasis and the inflammatory response in IBD [52]. The activation of FOXP3 + CD4+ regulatory T cells (Tregs) depends on TGF-β1 and IL-10. An imbalance in these cytokines contributes to the development of IBD [53]. Enterocolitis was spontaneously developed in mice with deficient IL-10 pathways [54]. Impaired IL-10 signaling has been observed in infants that presented with granuloma-positive colitis. TGFβ1 knockout mice, on the other hand, developed extended multi-organ autoimmunity [55].

For instance, fucoidan, a sulfated polysaccharide found in brown seaweeds, has demonstrated significant anti-inflammatory effects by inhibiting the production of pro-inflammatory species such as cytokines and enzymes, as well as reducing the activity of nuclear factor-kappa B (NF-κB), a well-known master regulator of inflammation [56,57]. 

Studies have demonstrated that the total antioxidant capacities of fucoidan is strongly and positively correlated with their polyphenol content, while a weak correlation exists with its xylose content [58]. For the first time, the synergistic effect of fucoidan was assessed using carbohydrates and polyphenols as model mixtures based on the mixture effect [58]. This mixture effect showed a strong positive correlation with polyphenols, while its relationship with the fucose content was positive but moderate [58]. 

Furthermore, Yang et al. investigated the effect of fucoidan on the expression of iNOS in the RAW26417 macrophage cell line. It was found that fucoidan inhibited the release of iNOS. Moreover, the activation of nuclear factor kappa B (NF-κB) and activator protein 1 (AP-1) is an important step in the transcriptional activation of the iNOS gene. Therefore, fucoidan selectively inhibited AP-1 activation, which is necessary to produce iNOS in activated macrophages. The inhibitory effect of fucoidan on the activation of AP-1 is closely related to its anti-inflammatory effect [59]. NO is overproduced in iNOS-induced activated macrophages and plays an important role in severe inflammatory diseases, such as sepsis and arthritis [60].

In addition, fucoidan also demonstrated antioxidant effects against ROS upsurge, thereby protecting intestinal cells from oxidative damage [61]. The anti-inflammatory properties of fucoidan were thoroughly investigated, revealing that they interfere with inflammation by different mechanisms. Studies have revealed that fucoidan can effectively reduce the level of COX2 in chondrocytes, which contributes to its anti-inflammatory properties [62]. They have also been involved in the production of PEG2 and COX2 in different cell types [63]. The NF-κB signaling pathway was a target of fucoidan, and it was found that fucoidan can suppress the NF-κB signaling pathway in different animal models and cell types, and can also inhibit the phosphorylation of MAPK enzymes, including ERK, JNK, and p38, in various models of inflammation, cancer, and ischemia-reperfusion injury [64].

In clinical trials, a study investigated the effect of fucoidan and fucoxanthin on 24 non-alcoholic fatty liver patients for 24 weeks. The patients were divided into two groups, and the placebo group took three capsules per day containing 550 mg of powdered cellulose while the treatment group received three capsules per day of low molecular weight fucoidan and high stability fucoxanthin (LMF-HSFx) containing 275 mg LMF and 275 mg HSFx. The treatment significantly reduced the lipids in the liver and decreased hepatic stenosis and inflammation by modulation of the leptin/adiponectin axis and activation of the AMPK signaling pathway [65]. In another crossover-designed study, eight male and eight female participants were supplemented with 1 g/day of fucoidan or placebo for 2 weeks. In this study, fucoidan supplementation reduced oxidative stress and inflammation following high-intensity exercise. It also improved the immune response by boosting natural killer cell activity and increased the production of anti-inflammatory cytokines [66]. Pulmonary functions were improved in patients with asthma after they received oligo-fucoidan supplementation [67]. In a clinical study, wheat peptide was combined with fucoidan (WPF) to treat patients with chronic gastritis once daily for 45 days. There were 53 individuals in the placebo group and 53 individuals in the treatment group. The study findings revealed that the WPF effectively improved chronic superficial gastritis and induced changes in the gut microbial composition [68]. 

Another beneficial compound derived from marine algae is alginate, which possesses the ability to form a gel-like substance that can coat the gastrointestinal lining. This protective barrier on mucosae may help to reduce irritation and inflammation in the intestines, providing symptomatic relief for IBD patients [69]. Moreover, the prebiotic effects of seaweed polysaccharides can promote a healthy gut microbiota, which is crucial for maintaining intestinal comfort and modulating immune responses [70].

### 4.2. NF-κB Pathway in IBD

The redox-sensitive transcription factor NF-ĸB is crucial in modulating the immune response associated with inflammatory reactions in IBD. Numerous stimuli activate NF-ĸB, including cytokines, oxidative stress, and microbial products [71]. p65/RelA, c-Rel, RelB, p50, and p52 are the five members that form NF-ĸB, while p105 and p100 are the precursors for producing this unit. During the cell activation, NF-ĸB becomes free to accumulate in the nucleus and activate gene transcription; in its stable state, NF-ĸB dimers are inactive due to binding to its inhibitor [72]. NF-ĸB activation contributes to the development and maintenance of intestinal inflammation by providing epithelial and immune cells with the capacity to protect the gut from pathogens and promote inflammation. The identification of microbes by intestinal epithelial cells involves pattern recognition receptors, such as NOD, TLRs, and NLRs, which activate NF-ĸB. *NOD2* gene mutations can cause a potent NF-ĸB activation, leading to an undermined host defense and damage to mucosal tissues. The loss of tolerance to commensal bacteria, which can result from disruption of the epithelial layer, can lead to an up-regulation of pro-inflammatory NF-ĸB [73]. IBD patients have been shown to have high levels of NF-κB, and strategies targeting the NF-κB pathway have the potential to be a therapeutic target in IBD [74].

### 4.3. Oxidative Stress Markers in IBD

Abnormal intestinal cell membranes, caused by an imbalance in the ratio of oxidative reactions to antioxidant defenses, have been demonstrated in patients with IBD [75]. Additionally, reactive nitrogen species (RNS), ROS, and ROS byproducts are commonly identified as secondary messengers that can provoke the activation of specific transcription factors, such as NF-ĸB, which constitutes an initial event leading to the transcription of a cascade of pro-inflammatory and pro-apoptotic genes [76,77]. Cellular differentiation, apoptosis, the immune response, and protein phosphorylation are just a few examples of ROS-dependent processes [78]. 

These substances may have an unpaired electron in their last valence shell such as the superoxide anion radical (O_2_•^−^) and the hydroxyl radical (OH•), but they may not be radicals and still have high oxidative potential, such as hydrogen peroxide (H_2_O_2_) [79]. When there is an increase in the production of these metabolites, harmful effects on important cellular structures (proteins, lipids, nucleic acids) begin to emerge [80]. In addition to endogenous production, the organism can be affected by exogenous ROS through environmental exposure. Among the endogenous defenses, we can mention superoxide dismutase (SOD), catalase (CAT), and glutathione peroxidase (GPx), which are classified as enzymatic components, and lipoic acid, uric acid, and bilirubin, which are classified as non-enzymatic; among the exogenous antioxidants, we can include carotenoids, vitamin E, vitamin A, and vitamin C [81]. 

### 4.4. Trefoil Factor 3 (TFF3)

TFF3, also named intestinal trefoil factor, is a mucin-binding peptide secreted by the goblet cells [82]. It has been found to increase at the site of mucosal injury and plays a critical role in the maintenance and integrity of the mucosal damage. In patients with UC, it was suggested that the level of TFF3 can predict mucosal healing and the disease activity. In fact, the TFF3 level increased in the damaged area [83]. Moreover, a strong correlation exists between the CRP and TFF3 in UC patients [84]. Therefore, TFF3 may be a valuable non-invasive marker to evaluate, monitor, and predict the disease’s activity and the rate of mucosal healing [85].

### 4.5. Lactoferrin

Another marker related to IBD is fecal lactoferrin. This protein increases in the stool during inflammation because of neutrophil infiltration into the intestine [86]. A single stool sample could be sufficient to measure this protein in the stool using an enzyme-linked immunosorbent assay (ELISA). The concentration of lactoferrin increases several hundred times in IBD patients compared with healthy individuals [87]. The fecal lactoferrin levels are 85–90% higher in active IBD patients compared to those with inactive IBD [88]. Studies have shown that fecal lactoferrin and calprotectin are similarly useful in assessing IBD activity, as fecal lactoferrin correlates with histological inflammation and calprotectin is associated with colonic inflammation at endoscopy [89].

### 4.6. SIRT1

SIRT1 is a master metabolic regulator, and the downregulation of SIRT1 was demonstrated in IBD patients with both CD and UC compared with healthy individuals [90]. Inflammatory cytokines, like TNF-α and IL-21, negatively regulate the expression of the SIRT1 [91]. Otherwise, the activation of SIRT1 by Cay10591 reduces NF-kB activation and inhibits inflammatory cytokine synthesis in IBD, while Ex527, an inhibitor of SIRT1, increases inflammatory cytokine production. 

Cay10591 treatment prevents and cures experimental colitis, while Ex527 exacerbates the disease by modulating the T cell-derived cytokine response. These findings suggest that SIRT1 activators could be beneficial for treating IBD [91].

## 5. Marine Ecosystem: A Useful Tool in the Management of IBD Symptomatology

Natural compounds have garnered interest in the management of IBD due to their multiple biological activities. Plant-derived compounds like curcumin from turmeric, polyphenols from green tea, and omega-3 fatty acids from fish oil have shown promise in reducing inflammation and supporting gastrointestinal health in IBD patients. Research continues to explore their therapeutic potential as complementary or alternative treatments to conventional medications.

In this wide context, the marine plant ecology may also be a source of novel bioactive chemicals that may have beneficial outcomes on IBD [92]. Both macro- and micro-algae generate an endless variety of chemicals, many of which are advantageous to human health. They have the power to improve our microbiota, reduce inflammation, and ease pain [93]. However, many algae are still unstudied, and their potential health benefits are undiscovered. In this review, we will examine the recently published research that has used marine algae or their extracts to provide potential treatments of IBD.

### 5.1. Focusing on Marine Algae in the Treatment of IBD

#### 5.1.1. Macroalgae

Macroalgae and their components have been intensively investigated as potential drugs in the treatment of IBD for years. In this section, we will discuss the studies related to macroalgae and their components in the treatment of IBD. 

The lipopolysaccharide extract of the common green alga *Ulva pertusa* was used in research by Son and colleagues to treat a mouse model of IBD induced by sodium dextran sulfate (DSS). After taking this substance orally, blood and colon levels of pro-inflammatory factors such as NF-kB and MAPK were decreased, as well as those of IL-4, IL-6, and IL-10 [94]. Polysaccharides extracted from the marine red alga Digenea simplex can improve carrageenan-induced edema, reduce inflammation induced by histamine, 5-hydroxytryptamine, and bradykinin, and inhibit the transfer of neutrophils to the peritoneal cavity and paws of mice. Testing peritoneal fluid showed that the concentrations of TNF-α and IL-1 β were also inhibited, indicating that polysaccharides can exert anti-inflammatory effects by inhibiting the expression of pro-inflammatory factors [95]. Sulfated polysaccharides extracted from the marine green alga *Caulerpa mexicana* (Cm-SPs) have anti-inflammatory and analgesic effects, and a paw edema model induced by dextran and histamine was inhibited by Cm-SPs 20 mg/kg, indicating histamine as a target of their anti-swelling effect. In addition, Cm-SPs reduce the paw edema induced by carrageenan, and the activity of myeloperoxidase illustrates that Cm-SPs exert an anti-inflammatory effect [96]. In another study, the gastroprotective effect of Cm-SPs on ethanol-induced gastric damage was studied. Prostaglandin and oxidative stress pathways were involved in this protection [97].

Additionally, *Ulva pertusa* extract has been shown to have a protective impact on tissue morphology; in fact, therapy with this extract restores the colon tissue architecture as well as tight junctions like occludin, claudin-1, and mucin. The direct impact that algae have on gut homeostasis, which is a major contributing factor to IBD, should therefore not be undervalued. Moreover, *Ulva pertusa* has restored the intestinal microbiota that was changed when DSS was administered [94]. Another study examined the potential of two compounds extracted from fucoidan, a long-chain sulfated polysaccharide found in various species of brown algae [56]. The mouse model of colitis was induced by DSS, and the treatment lasted 7 days; several parameters were examined such as weight loss, diarrhea, blood in the stool, the morphology of colon tissues, and the presence of markers of inflammation. The treatments were performed both by intraperitoneal and oral administration. It is important to note that, between the two routes of administration, the one that obtained the best overall result was the oral one, advancing that a possible treatment for IBD can be better conveyed through the oral route of administration. Due to limited absorption and tissue distribution of the parent molecule, the route of administration might considerably influence the effectiveness of the treatment given the large size of fucoidan, which ranges from 5 to 1000 kDa. Given that, oral fucoidan is likely to reach the target area in IBD [56]. Lean and colleagues’ findings suggest that fucoidan–polyphenol complex (Maritech Synergy) and depyrogenated fucoidan (DPF) can considerably lessen the pathophysiology linked to DSS-induced acute colitis. Considering that the response to colitis therapy depends on several variables, such as the location of the disease, the kind of inflammation, the pathogenic pathways, and the combination of numerous cytokines, it is necessary to find a drug or a chemical that can be effective on multiple targets [56]. 

Another brown seaweed with marked anti-inflammatory properties is *Dictyopteris undulata*, from which it is possible to extract zonarol. This compound belongs to the class of sesquiterpene hydroquinone, which has antioxidant and algicide properties, and inhibitory effects on phospholipase [98]. Yamada and colleagues examined zonarol at doses of 10 and 20 mg/kg in a mouse model of UC caused by DSS, utilizing 5-amino salicylic acid (5-ASA)-treated mice as a positive control, with a dose of 50 mg/kg. The findings demonstrated that 5-ASA and zonarol both significantly reduced the disease’s activity index, the duration of colic ulcers, and the amount of inflammatory mucous membrane infiltration, particularly by macrophages. Zonarol also stopped intestinal epithelial cells from dying and blocked the production of pro-inflammatory signaling molecules. In addition, in the RAW264.7 murine macrophage cell line, the cells were incubated for 24 h with different concentrations of zonarol (0, 1, 2, and 5 µM), and then an MTT assay was performed. The results indicated that zonarol did not affect the viability of the cells at the used concentrations. After that, LPS (10 mg/mL) was administered either alone or in combination with different concentrations of zonarol, which did not significantly affect the cell viability. Zonarol prevented activation brought on by lipopolysaccharide LPS [98]. Zonarol suppressed the expression of pro-inflammatory signaling molecules both locally (in the colon) and systemically (in serum), including TNF-α and IL-6. Both a heightened immunological response and a decrease in inflammation are facilitated by this action. It is important to emphasize the importance of blocking apoptosis, since excessive epithelial cell death might weaken the intestinal barrier and make it easier for bacteria and other dangerous substances to invade [98]. According to one study’s findings, zonarol is a useful marine bioproduct that, like sulfasalazine, a common drug used to treat ulcerative colitis, may protect against experimental ulcerative colitis by inhibiting both inflammation and apoptosis [98,99]. 

Sudirman and colleagues tested another polysaccharide extract from the red macroalga *Eucheuma cottonii* (EC) on the inflammatory response and DSS-induced colon damage in a mouse model of colitis [100]. The study’s technique comprised giving mice DSS for seven days to induce colitis, then treating them with several dosages of EC extract and, as a positive control, Curcumin. EC extracts were given to male BALB/c mice, which prevented them from losing weight and decreased the colon’s weight/length ratio while also raising IL-10 levels and reducing the production of pro-inflammatory cytokines. TNF-α, IL-1β, and IL-6 were shown to be lowered in blood levels by EC extract and Curcumin, according to cytokine analysis. Histopathology revealed that, after inducing colitis by DSS, EC extract and Curcumin decreased inflammatory cell infiltration and restored the integrity of the colon walls [100]. Sudirman et al. evaluated the toxicity of EC’s polysaccharide extract using RAW 264.7 macrophage cells. This was accomplished via a cell viability test (MTT), which assessed the cells’ capacity to endure following treatment with various EC extract doses [100]. The EC extract led to a reduction in the activity index of the disease, which is a widely used indicator to validate the severity of colitis in mice. 

The study conducted in 2016 by Brito et al. focused on a sulfur polysaccharide that was taken out of the seaweed *Hypnea musciformis* and its capacity to prevent intestinal damage in rats caused by trinitrobenzene sulfonic acid (TNBS) [101]. Sulfur polysaccharide (PLS) was described and its preventive potential against colitis in animals was assessed by researchers. Oral PLS administration reduced dose-dependent signs of colitis, both macroscopically and microscopically. Biochemical indicators of inflammation, including glutathione, malondialdehyde, myeloperoxidase, nitrates/nitrites (NO3−/NO2), and cytokines, were also investigated in the study. According to the findings, PLS lowers the levels of pro-inflammatory cytokines, free radical generation, and neutrophil infiltration. This implies that PLS has antioxidant and anti-inflammatory properties. Histologically, PLS helped decrease cryptic abscesses, inflammatory cell infiltration, colon mucosal architecture degradation, and even goblet cell depletion—a well-known sign of IBD [101]. 

Ryan et al. investigated the effects of β-glucans generated from *Saccharomyces cerevisiae* and seaweed belonging to the family Laminaria (*Laminaria hyperborea* and *Laminaria digitata*) on the inflammatory pathway mediated by IL-17 in pig colons [102].

The production of cytokines linked to Th17 (IL-17a, IL-17F, and IL-22), the IL23R receptor, and IL-6 was considerably decreased by the tested β-glucans. The Foxp3 transcription factor linked to TGF-β or TREG was not found to be expressed differently; however, there was a notable decrease in IL-10 [102]. In the pig colon, these β-glucans decrease Th17 cells’ immune responses by lowering the production of important cytokines while leaving the TREG pathways intact. The “switch” factors that are known to direct the immune response toward Th17 or TREG, such as TGF-β and IL-6, are not altered by this activity. Thus, β-glucans show promise as modulatory agents of intestinal inflammation, which may be important for IBDs [102]. 

Ardizzone et al. investigated the effect and the mechanism of *Ulva pertusa* for DNBS-induced colitis. It was shown that the action of *Ulva pertusa* on the SIRT1/Nrf2 axis improved the antioxidant response and the modulation of the apoptosis pathway induced by colitis, regulating the expression of p53, Bax, Bcl-2, and caspases [103]. 

The low molecular-weight sulfate ulva polysaccharide (LMW-ulvan) showed a protective effect against colitis induced by DSS through the modulation of oxidative stress and inflammation [104]. 

Moreover, ulvan extracts deriving from *Ulva Lactuca* exhibited noticeable vascular anti-inflammatory properties by decreasing TNF-α and IL-1 levels [105]. 

The host’s immune responses play a critical role in regulating mucosal immunity and integrity against IBD. From this point of view, *Ulva pertusa* showed an immunomodulatory activity associated with TLR4 and NLRP3 inflammasome modulation [106]. 

Additionally, Mingfeng Ma et al. reported that polysaccharides from the edible alga Enteromorpha clathrata were able to compensate for body weight loss, reduce the chance of bleeding, improve stool consistency, and improve mucosal damage in diseased mice, as well as amplifying the abundance of the gut microbiota Parabacteroides spp. [107]. 

The combination of *Laminaria japonica* (LJ), an edible seaweed, and a probiotic demonstrated a synergistic effect observed by the modulation of IL-1β, IL-6, and IL-12 (P40). According to these findings, LJ worked in concert with probiotics to effectively induce protection against colitis in mice [108]. 

The anti-inflammatory properties of a methanolic extract of the green marine algae *Caulerpa mexicana* were investigated by Bitencourt et al [109]. The extract effectively improved the reduction in body weight and the clinical manifestations of colitis, significantly ameliorated the clinical signs observed in UC, and decreased cytokine levels from the Th1 (IFN-γ, IL-12, and TNF-α) and Th17 (IL-6 and IL-17) immune response pattern. This decrease could be linked with a reduction in the colonic tissue damage observed in the colon of the mice receiving DSS [109]. Regarding anti-inflammatory effects, sulfated polysaccharides extracted from the marine green alga *Caulerpa mexicana* showed anti-inflammatory and analgesic effects by interfering with hemoxigenase-1 [110].

#### 5.1.2. Microalgae

*Chlorella vulgaris* (CV) was examined in the treatment of mice’s colitis. Weight loss, constipation, and rectal bleeding were among the symptoms of colitis that were markedly reduced by CV supplementation. Improved mucosal damage and less inflammatory cell infiltration in the colon were demonstrated by histological investigation. Furthermore, CV therapy raised the proportion of regulatory T cells (Tregs) in the mesenteric lymph nodes and spleen, pointing to a possible immunological regulatory mechanism that alleviates colitis [111]. *Spirulina platensis* (SP) and *Dunaliella salina* (DS) were investigated on acetic acid-induced colon inflammation in rats, and both microalgae demonstrated anti-inflammatory properties by inhibiting the production of proinflammatory cytokines such as IL-1β, TNF-α, and IL-6. Lipid peroxidation and oxidative stress markers were investigated, and it was found that SP and DS decreased the levels of malondialdehyde (MDA) and protein carbonyl (PCO), increased the levels of GSH, catalase (CAT), and superoxide dismutase (SOD), and modulated the immune system through interacting with immune cells and affecting cytokines that help to balance the immune function [112].

All data related to the studies discussed in this section are summarized in Table 1 and Table 2. 

## 6. Pharmacokinetic Properties of Marine Algae-Derived Compounds

Marine organisms play a pivotal role in the search for innovative compounds that can be developed into drugs. Pharmacokinetics studies show how these marine algae-derived agents are absorbed, distributed, metabolized, and excreted from the body. In addition, pharmacokinetic parameters can be studied, such as the half-life (t1/2), clearance (CL), the area under the curve (AUC), maximum concentration (Cmax), time to reach maximum concentration (Tmax), volume of distribution (Vd), and bioavailability (F). All these criteria help determine the appropriate doses and timing to achieve the best therapeutic effect and minimize the potential side effects [114]. The pharmacokinetics and some pharmacodynamic parameters of compounds derived from marine sources have been investigated in several studies. In this section, we will focus on marine algae-derived bioactive compounds. 

**Figure 2:**
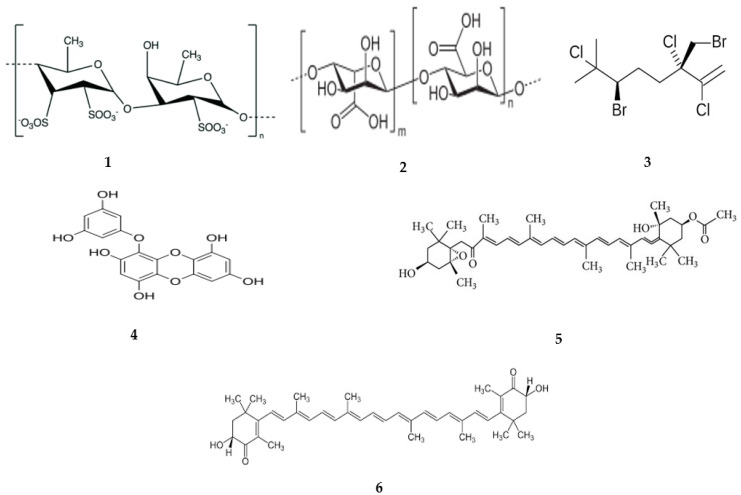
The chemical structure of marine algae-derived compounds: (**1**) fucoidan; (**2**) alginic acid; (**3**) halomon; (**4**) eckol; (**5**) fucoxanthin; (**6**) astaxanthin.

Fucoidan (Figure 2(**1**)) is a sulfated polysaccharide found in various types of algae, and it has been proven to possess a wide range of pharmacological activities, such as anti-diabetic, anti-viral, anti-cancer, anti-coagulant, and immune-supporting properties [115]. The pharmacokinetics of fucoidan were studied in various animal models such as mice, rabbits, and rats. When it was administered via IV, fucoidan (7.1 kDa) isolated from *Laminaria japonica* quickly appeared in the plasma, with a peak concentration of 110.53 µg/mL reached within 5 min. The serum concentration–time profile follows two exponential patterns, the initial phase with a short half-life (11.24 ± 2.93 min) and the final phase with a long half-life (98.20 ± 25.78 min). Conversely, fucoidan was detected in the rabbit serum 2 h after 200 mg/kg oral administration [116]. Higher molecular-weight fucoidan 107.8 kDa from Fucus vesiculosus showed a peak plasma concentration of 66.37 μg/mg and an area under the curve (AUC) of 198.11 μg/g·h after IV administration in mice. The absorption of fucoidan is influenced by its molecular weight, and lower molecular-weight fucoidan is absorbed better than higher molecular-weight fucoidan after oral administration in rats [117]. The mean residence times (MRT) in the blood were similar between the IV and oral administration of fucoidan in rats. High molecular-weight fucoidan has shown a long-term MRT presence in the blood and accumulation in organs such as the spleen, kidneys, and liver [117]. Fucoidan is initially concentrated in the skin after topical application and, over time, it is distributed to the other parts of the body such as the muscles and plasma [118]. Fucoidan was detected in the small intestine and jejunum epithelial cells when the rats were given a diet containing fucoidan. A study conducted by Suda et al. found the presence of fucoidan in the liver macrophages, indicating metabolic degradation by these types of cells [119]. The clearance rate of fucoidan in rats was higher when it was administered orally (0.138 mg/μg/mL/h) than for intravenous administration (0.0037 mg/μg/mL/h) [120]. After topical application in rats, fucoidan showed a prolonged half-life in the plasma and low bioavailability [118].

Extensive research in animals has been conducted to investigate the pharmacokinetics properties of fucoidan. These studies have shown that high molecular-weight fucoidan is absorbed after being administered orally or transdermally and distributed throughout different tissues and organs. Several human studies have demonstrated the absorption of fucoidan after oral administration. In one study, 40 volunteers were given 3 grams of 75% sulfated galactofucan extracted from Undaria pinnatifida three times daily for 12 days. Fucoidan was detected in the plasma, with an oral bioavailability estimated to be less than 0.6% [121]. Another study in 10 healthy men found that 1 gram of 66 kDa fucoidan extracted from Cladosiphon okamuranus was detectable in the urine and serum within 3 h, with the levels increasing over 9 h. Larger studies involving 48 and 396 volunteers also confirmed the presence of fucoidan in urine after the oral consumption of *Okinawa mozuku* and *Nemacystus decipiens* seaweed, respectively [122,123]. 

Griffithsin (GRFT) is a lectin isolated from red algae that provides antiviral activity [124]. Pharmacokinetic studies in rats showed a dose-dependent serum concentration after IV administration at a dose (10–20 mg/kg), although it then declined rapidly. After 15 min of SC administration, GRTF started to appear and reached the Tmax in 4 h, indicating slow absorption. GRTF was undetectable in the serum 96 h after a single orally administered dose (20 mg/kg) [125]. The Cmax of GRFT in the plasma reached 0.32 µg/mL in mice and 0.46 µg/mL in guinea pigs on day 11, following 14 days of daily SC injections of 10 mg/kg of GRTF [126]. After the SC injection of GRFT, the volume of distribution declined with increasing does. The research showed that GRFT administration after SC injection tends to accumulate primarily in the spleen and then distributed to other organs such as the liver and kidney after multiple doses. It is recommended to monitor the potential immune response resulting from GRFT immune-related toxicity [126]. After IV, SC, and oral administration in rats, GRFT was detected in an unchanged form in the urine and feces, and the bioavailability of GRFT was dose-dependent after SC injection, for 10 mg/kg it was 43%, and for 20 mg/kg it was 90% [125].

Alginates (Figure 2(**2**)) are polysaccharides extracted from brown algae and are used in pharmaceutical formulations as a drug carrier. Alginate oligosaccharides were measured in the plasma of mice after oral administration with Cmax 24.5 µg/mL of Cmax and Tmax for 5 min, and less than 5% were not absorbed. Alginate showed a two-phase plasma concentration–time profile after IV administration. After IP injection, the Cmax in plasma appeared after 6 h [127]. After IP and SC administration in mice, sodium alginate was detected in the spleen, liver, and kidney [128]. Two hours after oral administration, alginate oligosaccharides were not detected in mice’s plasma, indicating it is not reabsorbed by the renal tubules [127]. The t1/2 of sodium alginate was 12.5 h after IP injection in mice, its bioavailability was 44% and 5%, and it was excreted in urine after IP and SC administration [128]. 

Halomon (Figure 2(**3**)) is a halogenated monoterpene extracted from the red alga *Portieria hornemannii* that has an anticancer effect. Pharmacokinetic investigations in mice showed rapid distribution after IV administration. There is a difference in plasma concentration between males and females after 5 min of halomon administration, with a moderate bioavailability of 45% following SC injection and a low bioavailability of 4% after oral administration [129]. It was found that halomon was rapidly absorbed and distributed to different tissues such as in the brain, kidneys, lungs, and fat after IV administration in mice, given that it can cross the blood–brain barrier [129]. In vitro, hepatic cytochrome p-450 enzymes in humans and mice were responsible for the metabolism of halomon by removing chlorine and bromine atoms. Halomon was eliminated with a clearance of 36–56 mL/min/kg after IV injections in mice and the bioavailability was 45%, 47%, and 4% after IP, SC, and oral administration, respectively. Halomon was eliminated mainly through the biliary way or hepatic metabolism [129]. 

Eckol (Figure 2(**4**)) is a phlorotannin derived from red and brown algae and has different therapeutic potentials. No pharmacokinetic data were available in animals, but in silico analysis indicated moderate intestinal absorption and high plasma protein binding [130].

Fucoxanthin (Figure 2(**5**)) is a carotenoid extract from algae and diatoms with several biological activities [131]. Pharmacokinetic examinations in animals illustrated a rapid metabolism of fucoxanthin to fucoxanthinol and amarouciaxanthin A after oral and IV administration. They were measured in the plasma after 1 h of oral administration with Tmax for 4 h, and the Cmax was doubled in fucoxanthinol compared with amarouciaxanthin A. Fucoxanthin and its metabolites were measured in various tissues such as the liver, lung, kidney, heart, and spleen after oral administration, and the volume of distribution of fucoxanthin was much smaller than its metabolites after IV administration [132]. In the gastrointestinal tract, fucoxanthin is metabolized by lipase and cholesterol esterase into fucoxanthinol and then absorbed by the intestine [133]. In the liver, fucoxanthinol is converted into amarouciaxanthin A by short-chain dehydrogenase/reductase [134]. The T1/2 of fucoxanthinol was 4.5 h and that of amarouciaxanthin A was 6.7 h after fucoxanthin was given orally in mice. Fucoxanthin was eliminated from the liver and kidney quickly compared with that in adipose tissue [132].

Astaxanthin (Figure 2(**6**)) is a pigment in bacteria, crustaceans, fish, and microalgae with different pharmacological activities such as anticancer, antidiabetic, and neuroprotection activities. Pharmacokinetic studies showed higher systemic availability after IV administration compared with oral administration. In rats given a diet containing 3% astaxanthin, which accumulated in the spleen, kidney, and adrenals, it was also highly distributed to all organs 8 and 24 h after the orally administered dose of 100 mg/kg in rats [135]. The clearance of astaxanthin was slower after IV injection at higher doses in rats and it was eliminated mainly through non-renal clearance [136]. 

In human studies, the pharmacokinetics of the semi-synthetic compound astaxanthin were demonstrated in healthy volunteers. It was measured in the plasma with a peak concentration of 1.3 mg/L at 6.7 h and the T1/2 varied between 11 and 33 h [137]. In another study conducted by Odeberg, the Cmax was 55 μg/L, with a half-life of 16.7 h [138]. The results of a study conducted on volunteers, smokers, and non-smokers revealed that smoking reduced astaxanthin’s elimination half-life and that meal consumption improved astaxanthin’s bioavailability by delaying absorption and reducing clearance [139]. 

Considering the valuable pharmacokinetics of marine algae-derived molecules, the research aiming to optimize the formulation of drugs for the delivery of active principles derived from algae is crucial in maximizing their therapeutic efficacy and ensuring targeted delivery. In this context, a recent article discussed the composition and production technology of fucoidan tablets, which were found to enhance the stability, bioavailability, and controlled release of fucoidan, ensuring that the active compounds reach the intended site of action in the intestines effectively, and thus demonstrating the potential for targeted intestinal delivery [140]. This approach could improve the therapeutic outcomes for IBD patients by directly addressing inflammation in the gut, reducing systemic side effects, and improving patient compliance through oral administration [140].

## 7. Conclusions

The exploration of marine algae- and marine-derived components for the treatment of IBD is promising in terms of both research progress and the development of new therapeutic strategies. As thoroughly indicated by the preclinical studies reported in this review, these precious marine resources have demonstrated significant anti-inflammatory, antioxidant, and immunomodulatory properties that are particularly beneficial in mitigating IBD. 

Based on these assumptions, significant advancements in IBD treatment are forecasted due to the emerging potential of marine algae-derived biomolecules to effectively reduce gut inflammation and oxidative stress, positioning them as promising natural therapeutics. Future predictions may include an increase in clinical trials assessing the efficacy of these compounds, leading to innovative treatment protocols that surpass current options. In particular, more extensive, rigorous, and long-term clinical trials are essential to establish standardized dosing regimens, safety profiles, and efficacy parameters.

To fully match this purpose, stronger interdisciplinary collaborations between marine scientists and medical researchers are in order to accelerate the integration of algae-based therapeutics into clinical practice. Furthermore, the pharmaceutical industry’s interest in developing algae-derived drugs to ultimately improve the quality of life of patients with IBD could open up new avenues for sustainable drug development, including the use of innovative nano-administration systems as well as the employment of innovative biopharmaceutical methods.

We also retain that future research should focus on several key areas to advance the understanding and application of seaweed-based therapies for IBD by performing mechanistic studies, bioavailability studies, and formulating insights, while also considering the concept of personalized medicine, since understanding individual variations in responses to seaweed-based treatments could lead to more effective therapeutic strategies for IBD patients. In conclusion, the integration of seaweed-derived components into the therapeutic landscape of IBD holds considerable promise. Continued research and development in this field could lead to innovative, natural treatments that complement existing medical approaches, ultimately improving the actual condition of individuals living with IBD.

## Figures and Tables

**Table 1:** Studies summarizing marine algae in in vitro cell models of IBD.

Type of Algae	Study Design and Concentrations	In Vitro Cell Type	Control	Main Finding	Mechanisms	Reference
Korean seaweeds, Ulva pertusa polysaccharide (UPP)	1.5 × 10^6^ cells/well of LS174T and Caco-2 was incubated for 24 h (5 % CO_2_, 37 °C). UUP (1, 10, and 100 μg/mL) were added to the cells and incubated for 24 h. Total mRNA was obtained for further analysis. Culture media was used as a negative control (NC), and propionic acid (5 mM) as a positive control (PC).	LS174T and Caco-2 cells	Culture media, propionic acid 5 mM	↑ mRNA expression of MUC2, ZO-1, occludin, and TFF3	Strength Tight junction (TJ) and proliferation and survival of the epithelial cells.	[94]
Fucoidan, a sulfated polysaccharide from brown algae	Caco-2 cells were seeded at 3.75 × 10^5^ cells/well, the culture media was changed every 3 days until the cells fully differentiated, and the cells were used in passage number 48–62. RAW264.7 cells were seeded at 8.5 × 10^6^ cells/well and were used in passage number 10–30, 1.5 fucoidan (500 µg/mL) was applied to the apical side for 3 h and LPS 100 ng·mL was added to the basolateral side and after additional 3 h, the basolateral supernatant was collected to be used for marker analysis.	co-cultured Caco-2 and RAW264.7	Budesonide 1 µM and TNF- α 50 µg/mL	↓ mRNA expression of IL-8, TNF-α	Inhibition of TNF-α secretion resulted from LPS-stimulated RAW246.7.	[113]
Zonarol extracted from the brown algae, *Dictyopteris undulata*	RAW246.7 cells were seeded at 1 × 10^5^ cell/cm^2^, and after 24 h LPS 10µg/mL was added with or without zonarol 2 µM, 24 h later the cells were subjected to the required assay.	RAW264.7	The cells not treated by LPS or zonarol were used as a normal control	↓ NO, IL-1b, IL-6 and iNOS	Inhibit the inflammatory reaction in the macrophage cell line	[98]

**Table 2:** Summary of in vivo studies analyzing marine algae properties in IBD.

Type of Algae	Study Design and Dose	Model	Inducer/Control	Main Findings	Mechanisms	Reference
Korean seaweeds, Ulva pertusa polysaccharide (UPP)	Oral prophylactic Administration of UPP at doses (50, 100, and 200 mg/kg) once daily for three weeks After UPP administration, a 5% dextran sulfate sodium (DSS) was administered for the next eight days to induce UC	Mouse model	Dextran sulfate sodium, mesalazine (100 mg/kg)	↑ IL-4, IL-10, and IgA, occludin, and claudin-1 ↓ IL-1β, TNF-α, iNOS, IL-6, MPO, ERK, and p38 phosphorylation	Interfere with MAPK and NF-κB signaling pathway leading to inhibition of the inflammatory response-associated signaling pathways.	[94]
Depyrogenated fucoidan (DPF) and fucoidan-polyphenol complex (Maritech Synergy) extracted from *Fucus vesiculosus*	UC was induced by using DSS in the drinking water from day 1 to day 8. then the treatment group received two fucoidan extracts for 7 days, either by oral Synergy or DPF) or intraperitoneal (DPF).	Male C57BL/6 mice	Dextran sulfate sodium/no positive control, the mice that didn’t receive SSD were a healthy control	IP DPF, ↓ −36.4% IL-1α, −28.6% IL-1β, −31.1% IL-10, −52.8% MIP-1α, −43.7% G-GSF, MCP-1 (%NA) and ↑ 58.6% IFN- γ, 115.8% RANTES. Oral DPF, ↓ −55.9% IL-1α, −51.2% IL-1β, −62.2% IL-10, −46.5% MIP-1α, −60.0% MIP-1β, −80.6% G-CSF and −51.0% GM-GSF. Oral Synergy, ↓ −71.3% IL-1α, −55.7% IL-1β, −50.7% IL-3, −69.2% IL-10, −26.5% IL-12(P40), −29.4% IL-12 (P70), −30.8% IL-13, −60.9% MIP-1α, −72.7% MIP-1β, −85.2% G-CSF and −51.9% GM-GSF, −36.6% Exotaxin, and −68.0% TNF- α.	Affect pro-inflammatory signaling and macrophage pathways such as p38, Erk, JNK, HMGB1, and NF-κB.	[56]
Hydroquinone zonarol from *Dictyopteris undulata* (brown algae)	2% of DSS was used in drinking water for 14 days and at the same time 5-aminosalicylic acid (5-ASA at a dose of 50 mg/kg, and/or zonarol at doses of 10 and 20 mg/kg were used orally once a day for 14 days.	Male Slc:ICR mice	Dextran sulfate sodium/5-ASA 50 mg/kg as a positive control	↓ 44.4% TNF-α, 15.2% IL-1β, 21.5% iNOS, 28.1%, TUNEL+	Inhibit the TNF-α signaling pathway.	[98]
polysaccharide-rich extract from *Eucheuma cottonii* (EC)	2.5% (*w*/*v*) DSS was used in drinking water for 7 days, at the same time, EC at the dose 0.35; 0.70; or 1.75 g/kg, and curcumin 0.10 g/kg was orally delivered once per day of DSS treatment for 7 days.	male BALB/c mice	dextran sulfate sodium/curcumin 0.10 g/kg as a positive control	↓ TNF-α, IL-1β, and IL-6 in serum	Block TNF-α signaling pathway.	[100]
sulfated polysaccharide (PLS) from *Hypnea musciformis*	The animals were pretreated orally with PLS (10, 30, and 60 mg/kg, for three days, or dexamethasone (1 mg/kg, s.c.) then trinitrobenzene sulfonic acid (TNBS) was administered as a single intracolonic	male Wistar rats	trinitrobenzene sulfonic acid (TNBS) in a 50% ethanol/Dexamethasone (1 mg/kg, s.c.) as a positive control	↑ 246.3% GSH ↓ 46.8% MDA, 220% NO_2_/NO_3_, 22.7% IL-1β, and 27.4% TNF-α	Inhibit the synthesis and release of the product of the Inflammation.	[101]
β-glucans derivedfrom seaweeds *Laminaria hyperborea* and *Laminariadigitata*	The animals were divided into 4 dietary groups (i) basal diet (BD), (ii) BD + β-glucans from Laminaria hyperborea, (iii) BD + β-glucans from Laminaria digitata, and (iv) BD + β-glucans from Saccharomyces cerevisiae.	Pigs	NA	↓ IL-17a, IL-17F, IL-22, IL-23R, IL-5, IL-6 in S. cerevisiae group. ↓ IL-17a, IL-22, IL-23R, IL-10, IL-6 in L. digitata group. ↓ IL-17a, IL-17F, IL-22, IL-23R, IL-6 in L. hyperborea group.	Modulate the Th17 pathways without affecting TREG pathways.	[102]
*Ulva pertusa* (green alga)	2,4,6-dinitrobenzene sulphonic acid (DNBS in 50% ethanol was administered intrarectal as a single dose to induce colitis. Then Ulva Pertusa extract was administered orally for 4 days.	male CD1 mice	2,4,6-dinitrobenzene sulphonic acid (DNBS)/the control group treated with saline	↓ nitrotyrosine, Bax/Bcl-2 ratio, mast cell infiltration, NF-κB, IL-5, IL-9, IL13, iNOS, COX2, P53, Bax, Caspase-3, Caspase-8, Caspase-9, MDA, ↑ IκBα, IL-4, Bcl-2, Nrf2, GSH, CAT, SOD, SIRT1, Mn-SOD, HO-1	Modulate Nrf2/SIRT1 and NF-κB pathway.	[103]
polysaccharide extracted from *Enteromorpha clathrate* (EPC)	dextran sulfate sodium (DSS) in 2.0% (*w*/*v*) in drinking water for 8 days, at the same time EPC at a dosage of 100 mg/kg/day was used orally.	male c57bl/6j mice	dextran sulfate sodium (DSS)/normal control group which serves as the baseline for comparison.	No markers evaluated in this study	N.A	[107]
*Ulva pertusa* (green alga)	2,4,6-dinitrobenzene sulphonic acid (DNBS in 50% ethanol was administered intrarectal as a single dose to induce colitis. Then Ulva Pertusa extract was administered orally for 4 days.	Male CD1 mice	2,4,6-dinitrobenzene sulphonic acid (DNBS)/the control group treated with saline	↓ ICAM)-1 and p-Selectin, CD68, CD4, CD8, TLR4, MYD88, TRAF6, NLRP3, ASC, Caspase-1 ↓ IL-6, IL-17, and IL-23 in the serum ↑ serum IL-10	Modulate TLR4/Myd88/TRAF6 Pathway and NLPR3 inflammasome.	[106]
Low Molecular Weight Sulfate Ulva Polysaccharide (LMW-ulvan) from Ulva pertusa	2% (*w*/*v*) DSS drinking water for 5 days, then LMW-ulvan at doses 50 and 100 mg/kg was administered orally for 7 days	Male C57BL/6 SPF mice	dextran sulfate sodium (DSS)/5-ASA (50 mg/kg)	↑ IL-4, CAT, GPx enzyme activity, occludin, ZO-1, Claudin-1↓ IL-1β, IFN-γ, and MDA in serum and colon	Suppress NLRP3 inflammasome activation, inhibit Th1 cell response, and enhance antioxidant defense system.	[104]
Laminaria japonica extract (LJE)	5% DSS in drinking water for 7 days, during the same time LJE was administered orally twice a day at doses of 100 and 300 mg/kg with probiotics at a dose of 300 mg/kg.	Male Balb/c mice	dextran sulfate sodium/normal animals were used as a control group	↓ IL-1β, IL-6, TNF-α, IL-17, IL-12 (P40)	Modulate Th17 and Th1/Th17 dependent pathway	[108]
*Chlorella vulgaris* (C.V) (green algae)	2.5% (*w*/*v*) DSS in drinking water for 7 days. Then C. vulgaris extract was orally administered 2 g/kg for 3 weeks	female C57BL/6 mice	dextran sulfate sodium/normal animals without treatment used as a control group	↑ Treg, short-chain fatty acid (SCFAs), absolute CD4 + Foxp3+, Rort + Foxp3+ regulatory cells, and CD4+ T cells in the spleen and mouse lymph node	Improve immune tolerance by immune regulatory mechanisms mediated by Tregs and modulate the microbiome.	[111]
Caulerpa mexicana extract (green algae)	3% DSS in drinking water for 14 days. During this period, the Caulerpa mexicana methanolic extract (2 mg/kg/day) was administered intravenously on alternate days	Male BALB/c mice	dextran sodium sulfate/control group received only saline without the DSS or the algae extract, serving as a baseline for comparison	↓ Th1, Th17, IL-6, IL-12, TNF-α, IFN-γ, and IL-17	Modulate Th1 and Th17 pathway.	[109]
*Spirulina platensis* (SP), *Dunaliella salina* (DS)	After sedating the animals by IP injection of pentobarbitone (35 mg/kg), acetic acid (4%, *v*/*v*) in saline was infused for 30 s into the colon to a distance of 8c cm using a polyethylene tube (2 mm) at the day 16, SP and DS was administered orally for 15 consecutive days with dose 500 mg/kg, in another group sulfasalazine was administered orally with dose 500 mg/kg at 13th, 14th, and 15th days.	Male Wistar albino rats	4% *v*/*v* acetic acid (AA)/normal control group received only saline, the group with a single dose of AA without treatment served as a positive control group, another group received sulfasalazine	↑ GSH, CAT, SOD ↓ MDA, PCO, MPO, TNF-α, IL-1β, IL-6, PEG2	Scavenge free radicals and decrease Infiltration of Inflammatory cells.	[112]
